# Emotional Labor in the Care Field and Empathy-enhancing Education by Reading Literature: A Brief Review

**Published:** 2018-08

**Authors:** Jonggab KIM

**Affiliations:** Dept. of English Language and Literature, Institute of Body and Culture, Konkuk University, Seoul, Republic of Korea

**Keywords:** Emotional labor, Depression, Deep acting, Surface acting, Empathy, Literature

## Abstract

**Background:**

Nursing is not just task-based work, but also emotional work. Nurses are also obliged to satisfy patients’ emotional needs, which often results in burnout and depression. We aimed to propose an effective method for reducing their emotional burden.

**Methods:**

We took theoretical measure to verify some theories on emotion and emotional labor.

**Results:**

Empathy can be enhanced by reading literature. It has been recognized from ancient times that reading is an empathic experience in its very essence. Reading is not possible without the reader identifying and sympathizing with the character in the story.

**Conclusion:**

Reading literature is not only an efficient means of enhancing empathy, but also very practical to implement. Among programs that proved efficient are role exchange programs, here-and-now spontaneity, perspective taking, simulation exercises, and so on. The problem with them is that they require special facilities and equipment. If they are not available, reading practice would be the best alternative.

## Introduction

Nurses are those who professionally look after patients and help their healing. To attend to such patients is extremely challenging and demanding, not only physically, but emotionally as well. Nursing is not just task-based work, but also emotional work (1). They are also obliged to satisfy patients’ emotional needs, which often results in burnout and depression (2). Such an emotional burden is one of the potential precursors to turnover (3). How to reduce their separation rate, not to mention their emotional hardship, is a perennial question. Empathy is regarded as one of the most effective methods in lightening emotional burdens.

Various problems of empathy education have been designed and implemented, but without much success (4). The difficulty with such education is due to the very nature of empathy: it is not teachable like cognition-oriented subjects (5). This paper proposes that empathy education should be more embodied and affective, and less cognitive. The cognitive process encourages the distinction between subject and object, but empathy is an emotion that undoes such a boundary. To use A.R. Hochschild’s term, deep acting in the care field is possible only when the boundary between nurse and patient is loosened (1). Otherwise, it becomes surface acting. Empathy is the human ability of one imaginatively becoming the other, which constitutes the reading experience.

How can we read literature without identifying with the character in the story? To put it another way, reading is deep acting par excellence, with the following implication that it is a powerful tool for enhancing empathy. However, such a value of reading literature has not been recognized by researchers of empathy education in the care field. Science is not empathic with literature. It is now high time that medical science is supplemented by the practice of reading literature.

This study aims to argue that reading practice is one of the most effective means of enhancing empathy.

## Results and Discussion

Many researches find that nurses who are empathic with patients feel less stress in their emotional labor. Empathy functions to reduce emotional suffering. Hence it needs to be investigated how emotion can become empathic. This is a theoretical question, for which literature review is essential. This study takes a theoretical methodology: it first defines what emotional labor is, and then discusses two theories of emotion and prove that even negative emotions can be turned into positive ones. After such a methodological preparation, we will proceed to demonstrate that it is not emotional labor itself that causes stress, but the absence of empathy.

### Emotional labor

Emotional labor is a term that Hochschild used in *The Managed Heart: The Commercialization of Feeling* to explain the emergence of new forms of labor in post-industrial society. Labor in industrial society was defined as a productive activity, that is, producing commodities for economic gain, and manual dexterity and tool manipulation were indispensable. Emotion was not associated with labor. However, the automatization of labor-intensive processes changed the status of emotion. Along with the rise of the service industry, emotion became labor in its own right. It does not produce commodities, but customer satisfaction. The waitresses in a restaurant not only serve food and beverages to customers, but do emotional labor, such as smiling, as well. They sell their smiles too. Of course, every smile is not labor. It becomes labor only when it is required as a necessary part of the job. To satisfy such emotional requirements, workers have to learn how to manage their feelings and expressions. It is expected that workers should not express their true feelings and hide their troubles with a smile. Emotional labor is involved in a wide variety of jobs, from flight attendant to health trainer and call center worker, and is also mandatory for nursing. Nursing in the 12^th^ century meant foster-mothering a young child. As mothers are to children, so nurses are to patients. Hochschild classified nursing as one of the highest emotional labors (1). But such an emotional requirement carries a heavy burden, leading to burnout, and even depression (2, 3). There is abundant evidence in the literature indicating that exhaustion due to the emotional labor performed by nurses gets worse and causes job dissatisfaction and burnout (2). Research based on interviews with nurses found that “emotional distress related to patient care, and fatigue and exhaustion” (3) pressed them to leave clinical practice.

But not all emotional labor always and necessarily leads to burnout and depression. “Service with a smile” (6), another definition of emotional labor, does not always exert the same adverse influences on the well-being of the workers. Emotional labor that causes enormous stress for one person can be experienced somewhat positively by another. There are varying degrees of emotional strain. Why are there such differences? In order to answer the question, Hochschild used the dramatological terms deep acting and surface acting (1).

Actors are those who play the roles of characters in a drama. They do not express their personal emotions, but those pertaining to the characters. Dramatic personae should not be confused with their off-stage characters. As actors, they enact or feign emotions. And well-trained actors fake their roles as if they were genuine. As they are deeply immersed in their given roles, they appear authentic and natural. It is only when they fail to be identified with the characters that their acting appears artificial and pretentious. Hochschild observed that nurses in the workplace display “fake” emotions in order to hide, control, or regulate their true emotions. However, such emotional labor, even faked in the beginning, can develop into deep acting, producing inner changes aligned with somatic modifications (7). Such deep acting often, if not always, contributes to high job satisfaction and high patient satisfaction. What is problematic and causes job-related stress, burn-out and depression (8) is surface acting. Surface actors try to alter their outward expressions, but their internal feelings are left intact (9), producing conflicts between outward expressions and internal feelings, and between mind and body. One smiles, but without the corresponding joy.

A lot of studies have demonstrated a positive relationship between surface acting and emotional exhaustion, depersonalization and low personal accomplishment (8, 9). Depersonalization is the phenomenon of the actor’s body becoming alienated from their mind. They act like robots with no inner feelings, detaching themselves from their emotions. Such forced acting brings about depression and exhaustion.

### Two theories of emotion

The difference between surface acting and deep acting reflects different relationships of mind and body. Deep actors do not experience mind and body conflicts. If acting is part of the job, it is needless to say that workers should turn surface acting into deep acting for their own well-being. Then, is it possible to transform faked emotions into true emotions? But before answering this question, a few words about the nature of emotion are necessary.

Antonio Damasio, one of the leading scientists of neuroscience, defined emotion as “actions or movements” of the body, “visible to others as they occur in the face, in the voice, in specific behaviors” (10). It is bodily movements that cause pleasure or displeasure on the most basic level. Emotions are not purely inner phenomena, but outward bodily manifestations. Otherwise, we could not discern each other’s emotions. And Damasio distinguished emotions from feeling in terms of their proximity either to the mind or to the body.

One of the major issues in the study of emotion is the causal relation between mind and body (11). One group of scientists approached emotion cognitively, emphasizing the function of the mind. Cognitive theory dictates that we smile because we register something and decode it as funny. The reason to smile gives rise to the facial movement. William Ickes explained that emotion is composed of three serial stages: decoding others’ emotions comes first, and then understanding their emotional states, and finally action (12). At the other pole of this cognitive theory is the somatic or affective theory of emotion, initially proposed by William James. This theory reverses the cognitive logic of emotion, contending that we smile first and then give a reason for it. Reasoning is produced after the event of bodily movement. Damasio, who sides with somatic theory, explained that feeling is an emotion that becomes conscious (10).

Between the two models of emotion, this paper contends that the affective or somatic one is more practical and more favorable to empathy education. According to cognitive theory, a nurse, for example, smiles because she is satisfied with what she is doing or because patients please her. The reason to smile comes first. No reason, no smile. If smiling is a job obligation, she has to force herself to make a fake smile, producing emotional strain. But the affective and somatic model tells a different story. Say that a nurse is obliged to fake a smile, even though she is in a bad mood. It appears unnatural and artificial at first. But since such an affective change or bodily movement is not alienated from the mind, it soon creates a reason to smile. She “came to feel the feeling appropriate to the emotion displayed. . . The expressions conjure up the feelings and the kinds of thoughts” (10). This means that a forced smile, made at first with conscious effort, can become a genuine smile.

### Empathy education and reading of literature

For our discussion it is important to notice that emotional labor can take the form of either surface acting or deep acting. If translated into an emotional term, deep acting has a close affinity with empathy (13). It was previously noted that an actor immersed in deep acting identifies with the character in a story. In such an act of identification the boundary between the actor and the character begins to disappear, the former becoming the latter: the actor feels as if he is the other. Philosophically speaking, such a feeling is defined as empathy. It was empathy with the weak and poor in particular that Adam Smith and David Hume regarded as the foundation of human society. If we don’t feel the suffering and distress of others as if they were our own, we will not be concerned about them or act on behalf of them. As such, empathy is built on a recognition of human weakness. “It is the weakness of the human being that makes us sociable, it is our common miseries that turn our hearts to humanity” (14).

At the core of empathy lies undoing the borderline between the self and the other, making them less distinct from each other. Such a liquidation of the distinction reduces the emotional burden of nurses in the care field. We observed that a smile becomes emotional labor if it is made in order to satisfy the emotional needs of the other. It is not if done for oneself. Empathy is exactly the momentum that makes a smile for the other become a smile for oneself. Then, the question is how to enhance empathy.

It has been observed that since empathy is not a cognitive skill, it “cannot be directly taught” (15). Empathy is not something to be learned by books or lectures, but is something to be felt and experienced. Which explains the difficulty of empathy education. It can only be facilitated by the right conditions and the necessary tools and resources. A few examples include role exchange programs, here-and-now spontaneity, perspective taking, and simulation exercises. A simulation program requires students to spend two days in a training center, including one overnight stay (16). Then, what if such facilities and time are not available? The answer is reading literature. This can be conducted in a normal setting without special equipment. The readers are invited to embark on an imaginative journey to empathy.

The therapeutic value of reading has been recognized since the middle of the 20^th^ century and institutionalized as bibliotherapy and literary therapy (17). Reading is in its very essence an an empathic experience. We cannot read without forgetting ourselves and identifying with the characters in a story. Citing Arthur Rimbaud’s famous words “Je est un autre,” Georges Poulet explained that such a transformation of the I to the other is exactly what reading performs. “Another I, who has replaced my own, and who will continue to do so as long as I read. Reading is just that” When I am absorbed in reading, a second self takes over, a self that thinks and feels for me. When I read Baudelaire or Racine, it is really Baudelaire or Racine who thinks, feels, and allows himself to be read within me”(18). It is a mistake if we take the phenomenon of “I becoming the other” as just a metaphor. Audre Lorde, a renowned poet and feminist who died of breast cancer at the age of 58, wrote *The Cancer Journals*, an autobiographical record of her struggle. “I’m not feeling very hopeful these days, about selfhood or anything else. I handle the outward motions of each day while pain fills me like a pus pocket and every touch threatens to breach the taut membrane that keeps it from flowing through and poisoning my whole existence” (19). It is not possible for us to read this passage without sympathizing with her. We feel her suffering as if it is ourselves who suffer cancer. If we are not moved, and still remain ourselves, it means that reading does not take place. The readers of Helen Keller’s *The Story of My Life* experience themselves as deaf and blind. And if we read Florence Nightingale’s *Cassandra*, we become nurses tending wounded soldiers. That is why Plato worried so much in *The Republic* about this transforming power of literature that he decided to drive poets out of his republic. His student Aristotle interpreted it positively, calling it catharsis, which inspired contemporary bibliotherapy to be born.

Such therapeutic value of literature has not been neglected by medical doctors and public health scholars. Patients not only suffer physical pains, but also narrate them: they have stories to tell. Such narratives invite readers to participate in their struggle with illness. Lorde’s *The Cancer Journals* is one example par excellence. Their strength with readers, even those who have never experienced any form of illness, can be explained by the universal condition of human weakness, as Rousseau described it. If we were immortal, it would be impossible for us to sympathize with those mortals who are doomed to suffer and die. Such a common fate of human weakness is the soil in which empathy grows. It is through stories that we exchange each other’s feelings, and through reading and listening. Such a recognition inspired Rita Charon to build a narrative medicine program at Stanford University, and to write *Narrative Medicine: Honoring the Stories of Illness* in 2006.

## Conclusion

It is true that nurses suffer from emotional labor, often causing their burnout. But it is also true that all emotional labor is not negative. Emotional labor is acting to manage feelings and expressions to satisfy the emotional requirements of a clinic. Hochschild distinguished deep acting from surface acting in terms of empathy with their role. What is important for this discussion is the fact that it is mostly surface acting, not deep acting, that produces depression and burnout. It follows that emotional strain for nurses will decline as their empathy ability is enhanced. Then, what is the best way to cultivate empathy? Empathy is a phenomenon in which the boundary between the subject and the other is undermined: I become the other. This paper argued that such a breakdown of the boundary cannot be achieved by cognition-oriented education. An empathy enhancement program should be implemented by somatic practices such as role exchange play. Another alternative is affective practice. It is the conclusion of this paper that reading literature is one of the most efficient means of educating about empathy, for reading begins only when the reader identifies with the character in the story. Reading enhances our capacity for empathy. One big advantage of reading over somatic practice is that it can be reinforced in normal settings, and does not require special equipment or facilities.

## Ethical considerations

Ethical issues (including plagiarism, informed consent, misconduct, data fabrication and/or falsification, double publication and/or submission, and redundancy) have been completely observed by authors.
